# Optimising an FFQ Using a Machine Learning Pipeline to teach an Efficient Nutrient Intake Predictive Model

**DOI:** 10.3390/nu12123789

**Published:** 2020-12-10

**Authors:** Nina Reščič, Tome Eftimov, Barbara Koroušić Seljak, Mitja Luštrek

**Affiliations:** 1 Department of Intelligent Systems, Jozef Stefan Institute, 1000 Ljubljana, Slovenia; mitja.lustrek@ijs.si; 2 Jožef Stefan International Postgraduate School, 1000 Ljubljana, Slovenia; 3 Computer Systems Department, Jožef Stefan Institute, 1000 Ljubljana, Slovenia; tome.eftimov@ijs.si (T.E.); barbara.korousic@ijs.si (B.K.S.)

**Keywords:** food frequency questionnaire, machine learning, supervised learning, feature selection, PROMETHEE, missing data, dimensionality reduction

## Abstract

Food frequency questionnaires (FFQs) are the most commonly selected tools in nutrition monitoring, as they are inexpensive, easily implemented and provide useful information regarding dietary intake. They are usually carefully drafted by experts from nutritional and/or medical fields and can be validated by using other dietary monitoring techniques. FFQs can get very extensive, which could indicate that some of the questions are less significant than others and could be omitted without losing too much information. In this paper, machine learning is used to explore how reducing the number of questions affects the predicted nutrient values and diet quality score. The paper addresses the problem of removing redundant questions and finding the best subset of questions in the Extended Short Form Food Frequency Questionnaire (ESFFFQ), developed as part of the H2020 project WellCo. Eight common machine-learning algorithms were compared on different subsets of questions by using the PROMETHEE method, which compares methods and subsets via multiple performance measures. According to the results, for some of the targets, specifically sugar intake, fiber intake and protein intake, a smaller subset of questions are sufficient to predict diet quality scores. Additionally, for smaller subsets of questions, machine-learning algorithms generally perform better than statistical methods for predicting intake and diet quality scores. The proposed method could therefore be useful for finding the most informative subsets of questions in other FFQs as well. This could help experts develop FFQs that provide the necessary information and are not overbearing for those answering.

## 1. Introduction

Adopting and maintaining healthy habits is extremely important and can at the same time be quite challenging. WellCo (http://wellco-project.eu) is an example of an EU-funded project whose aim is, among other objectives, to deliver a mobile application with a virtual coach that encourages its users towards healthier choices to improve their physical, cognitive, mental and social well-being. As proper dietary habits are very important for a healthy lifestyle, the application includes a module that monitors and encourages dietary habits. The essential part of the nutrition module is a food frequency questionnaire named the Extended Short Form Food Frequency Questionnaire (ESFFFQ), through which the virtual coach acquires information about the users’ dietary habits and returns recommendations based on the answers and quality scores calculated from them. The ESFFFQ [1] was developed by extending a validated questionnaire, the Short Form Food Frequency Questionnaire (SFFFQ), developed and validated by Cleghorin et al. [2], which calculates scores for fruit intake, vegetable intake, fish intake, sugar intake and fat intake, by adding food groups to cover additional targets—protein intake, fiber intake and salt intake. The base questionnaire, the SFFFQ, consists of 22 questions. An additional five questions were added to it in order to develop the ESFFFQ (see Table A1 in Appendix A). Although the ESFFFQ could still be considered short, the users might find answering the whole questionnaire in one take a bit overwhelming, especially since the users in the project are elderly and are expected to answer the questionnaire every two weeks. Consequently, determining which questions are beneficial and which could be removed without major loss turned out to be an important problem. To validate the questionnaire, the calculated nutrient values were compared with several validated questionnaires. The questionnaire, the ESFFFQ, is non-quantitative—it does not ask the user about the amount of consumed food, but about the consumption of food items in a certain time period. The list of questions (food items) is available in the Appendix A in Table A1. The scores are calculated based on the intake of nutrients provided by the consumed food and drink. The nutrient intakes are then transformed into quality scores with respect to the national dietary recommendations. The quality scores are discrete values—good, medium and bad. The development and validation of the ESFFFQ are more thoroughly described in our previous work [1], and this paper explores the performances of different methods used for selecting nutrient value and quality score-acquiring questions from a selected food frequency questionnaire.

FFQs are the most commonly selected tools in dietary monitoring, as they are efficient, cost-effective and non-invasive [3,4]. Once developed and validated, they are also quite simple to use. However, in order to include as many food items as is needed to cover the full variability of an individual’s diet, the questionnaires can get very extensive, which could be overwhelming for the user and could also lead to overestimating the predicted nutrients’ values [3]. Thoughtless removing of food items could, on the other hand, lead to underestimation, and it is important to find ways to optimize the lengths of questionnaires without losing validity.

The standard procedures for creating a food frequency questionnaire (FFQ) are time-consuming, and the selection of food items relies greatly on personal expertise; hence, an automated approach could be of great help. Gerdessen et al. [5] showed that by using mixed integer linear programming, the selection process becomes faster, more standardized and more transparent. However, the proposed method still relies on substantial expert knowledge in order to be used effectively. Machine learning could be used to help overcome the factor of personal expertise. Panaretos et al. [6] compared statistical and machine-learning methods for the evaluation of dietary patterns based on either foods or nutrients consumed; linear regression analysis was used to assess their associations with the cardiometabolic score. They showed that machine learning was superior to statistical methods and that it could be a valuable tool in the field of nutritional epidemiology. Machine-learning methods have also been used to detect dietary patterns [7,8] and to estimate nutrient values [9]; however, to the best of our knowledge no one has used machine learning to help experts find an optimal subset of FFQ questions without losing too much information.

The users included in the WellCo project are elderly and it was of great importance to find ways for them to answer as few questions as possible while still providing the system with sufficient information about their nutritional habits. Since they are supposed to answer questionnaires regarding other aspects of a healthy lifestyle as well, the number of questions could quickly become overwhelming. This problem could be overcome by finding a method to detect which questions in the ESFFFQ are really informative and which are redundant. Statistical methods, such as multiple imputation and zero imputation could, of course, be used to impute the answers to the questions the users did not answer, but it is reasonable to expect that at least zero imputation will underestimate the true intake and therefore return wrong diet quality scores. Besides, these methods, contrary to machine learning, are not designed to deal with intentionally missing answers.

Therefore, the paper proposes an approach that finds the questions that provide the most information, and estimates nutrient intake and diet quality scores from a subset of answers using machine learning. The hypothesis of the paper is that by using the proposed method, machine learning could better predict the nutrient intake and diet quality scores when using just the selected questions in comparison to statistical methods.

## 2. Material and Methods

### 2.1. Study Design and Population

In the WellCo project, the aim of nutrition monitoring was to find a way to ask elderly users as few questions about their dietary habits as possible and still get sufficient information to evaluate their nutrition.

The problem was explored as a set of single-target problems, which means that it was explored for each of the targets, diet quality scores, separately. The problem was explored for five of the targets—fat intake, sugar intake, fiber intake, protein intake and salt intake. The remaining three targets (fruit intake, vegetable intake and fish intake) are only dependent on one or two questions, and therefore the problem of reducing and ranking the features is trivial.

The answers to ESFFFQ were collected from 92 adults from Slovenia, Italy, Spain and Denmark as a part of the WellCo project and were then joined with answers from 197 adults included in SIMenu, the Slovenian EU Menu research project [10] aimed at collecting information about what and how much Slovenian inhabitants eat and drink. The FFQ in SIMenu actually included 104 questions that were organized into nine food groups (grains, diary, fruit, vegetables and potatoes, meat and fish and supplements, fat, sugar, drinks, other) and were asking the users about the frequencies of consumption of different food items. The possible answers (consumption frequencies) were equivalent to the answers available in the ESFFFQ. As the questions in the ESFFFQ were included in SIMenu, extracting the answers from SIMenu and adding them to the answers from the ESFFFQ was a very straightforward task.

Adults answering either of the questionnaires were between 18 and 80 years old. A more detailed description of the population in the SIMenu is available in the paper by Zupanič et al. [11].

### 2.2. Data Preprocessing

Input data consisted of answers to the questionnaire. The questions were of the multi-choice type, providing discrete answers. As not all of the questions had the same number of possible answers—and besides, some of the answers never contributed data—the answers were standardized to avoid one variable dominating others by subtracting the mean, and we scaled them to unit variance. Once normalized, the answers to questions were used as input features to machine-learning algorithms.

### 2.3. Methods

In this study, the performance of machine learning on different subsets of questions was explored for the following two types of machine-learning problems:

- **Regression problem**: In regression problems we try to predict continuous values. In our case, we try to predict the values of the intake of nutrient selected per food group.
- **Classification problem**: In classification problems the predicted values are discrete. In our case, we try to predict the diet quality scores for chosen nutrients and food groups.

For simplicity, the shorter expressions “amounts” and “scores” will be used when speaking of nutrient values’ amounts and diet quality scores respectively.

For comparison, besides machine-learning algorithms, the scores were also computed by using statistical methods. Statistical methods are commonly used as the state-of-the-art when dealing with missing questions, and could therefore be used for dealing with subsets of questions. First, the scores were calculated by using just the answers to the questions from a subset and setting the remaining questions to the frequency “rarely or never”. This is actually the so-called zero imputation method [12]. An alternative approach was also used, specifically multiple imputation [13,14]. The answers to the questions not included in the subset of questions are imputed with the multiple imputation with chained equations (MICE) method. These two methods were used as the baseline.

To calculate the scores, the amounts of nutrients were calculated based on users’ answers, and the amounts were further transformed to diet quality scores. For easier understanding, the example for target “vegetable intake” is presented. The amounts were calculated from sums of average amounts in Table 1 based on users’ answers (frequencies of consumption). Once the amount of intake is calculated, it is transformed to a diet quality score based on the recommendations listed in Table 2. For instance—a user answers that he/she eats a salad every day and that he eats vegetables 4–6 times per week. From Table 1 we calculate that the amount for vegetable intake is 176.8 g, and from Table 2 that the score for vegetable intake is 2 (medium).

In the field of machine learning, the expression features is used for the data that we feed to machine-learning methods. In our case, the features are the answers to the questions, and subsets of features represent answers to the questions included in the subset of questions.

#### 2.3.1. Dimensionality Reduction

**Figure 1:**
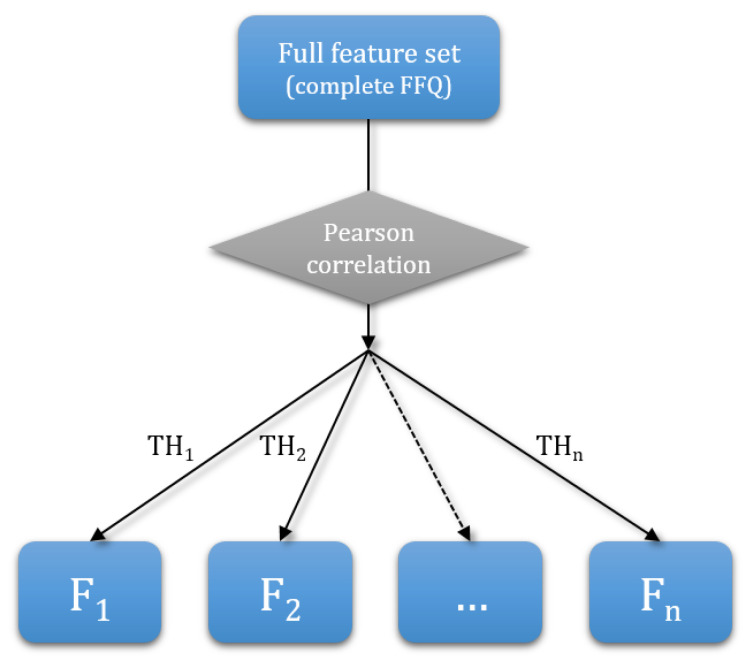
Pearson correlation coefficients.

The paper explores how reducing the number of questions in FFQs affects the diet quality scores, which could further be used to optimize the selection of the FFQ questions and shorten very extensive FFQs. First, Pearson correlation coefficients between the features were calculated. This approach was used as a Pearson correlation coefficient measures the statistical relationship between two variables. Features with a high correlation are more linearly dependent and therefore have a similar effect on the dependent variable. If two features are highly correlated, one of the two could be dropped. To get a subset of questions, we set a threshold TH_i_ and remove one of the two questions that have a correlation higher than this threshold. Thresholds were chosen experimentally. Pearson correlation coefficients were calculated and the thresholds were chosen in such way that number of elements in subsets determined by the thresholds fell approximately linearly. The set F_0_ represents the complete set of features. To produce the first subset of features F_1_, one of the two features whose correlation coefficient exceeded the chosen threshold was removed, and the same was done for every such pair of features. The procedure was repeated for the next thresholds. For thresholds TH_1_, TH_2_, …, TH_*n*_, we get subsets of features (questions) F_1_, F_2_, …, F_*n*_, which is shown in Figure 1. The thresholds and subsets obey the following relations:

$${\mathrm{TH}}_{1}>{\mathrm{TH}}_{2}>\ldots >{\mathrm{TH}}_{n}$$

$$F_{0}⊃F_{1}⊃F_{2}⊃\ldots ⊃F_{n}$$

#### 2.3.2. Machine-Learning Algorithms

Eight classification and eight regression models were built—some of them very simple, such as linear/logistic regression, decision tree and k-nearest neighbors; and the others more complex, including support vector machine, random forest, gradient boosting classifier/regressor, and finally, the voting classifier/regressor that combines all the previous models [15]. To build machine-learning models we used the sklearn library with default settings. Although tuning of hyperparameters—number of iterations, number of trees, max depth of trees, learning rate, etc.—could improve the performances of the models, it was decided not to do any tuning in order to have a fair comparison between the models. The goal of the paper was to find the subsets of questions on which most of the methods performed similarly or better than on the whole set of questions. Showing that machine-learning algorithms perform better on different subsets compared to the baseline models would be a desirable side product of the experiment.

#### 2.3.3. Evaluation Method—PROMETHEE

When dealing with a comparison of different models on different subsets of features, many evaluation measures can be used, and drawing clear summaries and conclusions from them may become a very challenging task. Besides, incorporating and comparing more different metrics makes the evaluation more robust and fair. We decided to combine different metrics by which models and subsets of features are compared. Specifically, the approach proposed by Eftimov and Kocev [16], which follows the idea of the preference ranking organization method for enrichment of evaluations (PROMETHEE) and was designed for multi-label classification problems was used. In the classification problem, the labels are the diet quality scores, and the regression problem predicts amounts, which are continuous values. The PROMETHEE methodology works as a ranking scheme, and we used it to rank the subsets of features based on the performances of different models trained on these subsets and to rank the methods on a subset.

For each subset of questions F_0_, F_1_, …, F_5_ (see Table 3), scores and amounts were predicted by using only the answers to the questions included in the subset. Next, the vectors of predicted values were compared with the vectors of the so-called true values, which were calculated from the full questionnaire, and different metrics—precision, which represents the percentage of positive identifications that are actually correct; recall, which represents the proportion of actual positives that were identified correctly; F-score, defined as the harmonic mean of the precision and recall; mean absolute error, which is the sum of absolute differences between our target and predicted variables etc.

**Figure 2:**
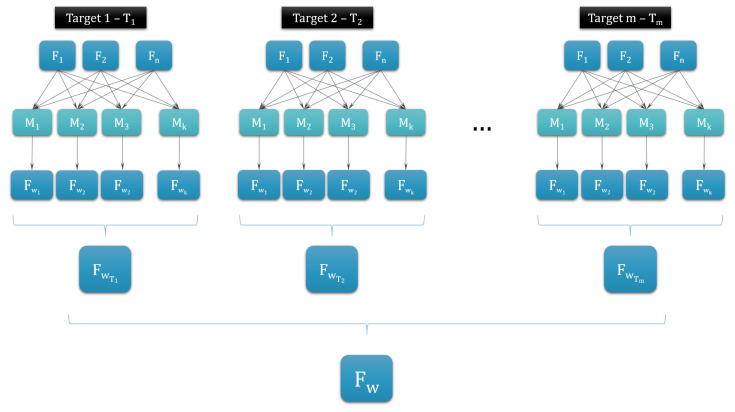
The pipeline of ranking the feature sets by using the PROMETHEE method. F represents a subset of questions, and M represents one of the chosen statistical or machine-learning methods. F_w*i*_ is the subset on which method $M_{i}$ performs the best, and F_w_T1__ is the subset on which most of the methods M perform the best. F_w_ is the subset of features on which methods perform the best for all targets.

The general pipeline of the evaluation method that returns the best feature subset for all targets (fat intake, sugar intake, etc.) is presented in Figure 2. The pipeline to extract the best algorithm is similar to the one presented in Figure 2, but with swapped labels for subsets (F) and models (M). The pipeline is explained for the case of ranking the methods.

First, *k* models are built with methods *M*_1_, *M*_2_, …, *M*_*k*_ on each of *n* feature subsets F_1_, F_2_, …, F_*n*_ with previously described dimensionality reduction. The models are built on all subsets for all *m* targets (fat intake, sugar intake, etc.) T_1_, T_2_, …, T_*m*_. For each target and each feature subset the decision matrix *D* is built. In *D*, $q_{j}\left(M_{i}\right)$ represents the performance measure of *j* for method *i*. In this paper, performance measure $q_{j}\left(M_{i}\right)$ is one of the calculated metrics, for instance, precision for the classification problem or mean square error (MSE) for the regression problem.

(1) $$D=[\begin{array}{cccc} q_{1}\left(M_{1}\right) & q_{2}\left(M_{1}\right) & \cdots & q_{N}\left(M_{1}\right)\\ q_{1}\left(M_{2}\right) & q_{2}\left(M_{2}\right) & \cdots & q_{N}\left(M_{2}\right)\\ \vdots & \vdots & \ddots & \vdots \\ q_{1}\left(M_{k}\right) & q_{2}\left(M_{k}\right) & \cdots & q_{N}\left(M_{k}\right) \end{array}]$$

The next step is to make pairwise comparisons between all methods for each performance measure. The preference function $p_{j}$ of a performance measure $q_{j}$ for two methods is defined as the degree of preference of one method over the other. For example, for methods *M*_1_ and *M*_2_ the *j*-th preference measure would be defined with the equation:

$$P_{j}\left(M_{1},M_{2}\right)=\left\{\begin{array}{cc} p_{j}\left(q_{j}\left(M_{1}\right)-q_{j}\left(M_{2}\right)\right) & q_{j}\;\mathrm{is}\;a\;\mathrm{maximizing}\;\mathrm{measure}\\ p_{j}\left(-\left(q_{j}\left(M_{1}\right)-q_{j}\left(M_{2}\right)\right)\right) & q_{j}\;\mathrm{is}\;a\;\mathrm{minimizing}\;\mathrm{measure}. \end{array}\right.$$

There are six types of preference function [17]. We chose the V-shaped preference function, which transforms the difference $d_{j}\left(M_{1},M_{2}\right)=q_{j}\left(M_{1}\right)-q_{j}\left(M_{2}\right)$ between the values of methods for the preference function using a linear function. The V-shaped function is defined as:

$$p\left(x\right)=\left\{\begin{array}{cc} 0, & x\leq 0\\ \frac{x}{Q}, & 0\leq x\leq Q\\ 1, & x>Q, \end{array}\right.$$

where *Q* is the indifference threshold, the greatest amount of difference that is insignificant.

After calculating preference measures $P_{j}$ for all pairs, the next step is to calculate the average preference index:

$$\pi \left(M_{1},M_{2}\right)=\frac{1}{N}\sum_{j=1}^{N}\omega_{j}P_{j}\left(M_{1},M_{2}\right).$$

where $\omega_{j}$ represents the relative significance (weight) of the performance measure $q_{j}$. For a more detailed explanation on how to calculate the weights, refer to the paper from Eftimov and Kocev [16].

The final step is to compute the positive, the negative and the net preference flows. The positive preference flow for $M_{i}$ quantifies how model $M_{i}$ outperforms other models and is calculated as:

$$\phi \left(M_{i}^{+}\right)=\frac{1}{n-1}\sum_{x\in M}\pi \left(M_{i},x\right).$$

The negative preference flow quantifies how method $M_{i}$ is outperformed in another way and is calculated as:

$$\phi \left(M_{i}^{-}\right)=\frac{1}{n-1}\sum_{x\in M}\pi \left(x,M_{i}\right).$$

Finally, the positive and negative flow are aggregated into net flow by subtracting negative flow from positive flow:

$$\phi \left(M_{i}\right)=\phi \left(M_{i}^{+}\right)-\phi \left(M_{i}^{-}\right).$$

The higher the net flow for method $M_{i}$, the better the overall performance of this method. By ordering the net flows in decreasing order we can get the rank of the feature sets for each target and each method. Next, the ranks are averaged and these averages are then ranked. This returns the ranking of the feature sets for each target. From here the most optimal method for one feature set can be deduced. We repeat a similar procedure for each target (fat intake, sugar intake …). From there we can get the most optimal method for all subsets.

To get the most optimal subset for each method, the matrix *D* was formatted a bit differently. For example, for method $M_{1}$ the rows of the matrix *D* represent the performance measures on feature sets—the first row presents the performance on the feature set F_0_, in the second row the performance measures on the feature set F_1_, etc. The above-listed equations were adjusted. This returned the most optimal feature set for method $M_{1}$, and the same procedure was repeated for all methods: *M*_1_, *M*_2_, …, *M_k_*. The pipeline for this example, on how to get the most optimal feature set F_w_T*i*__ for target *i* and the best feature set F_w_ for all targets is shown in Figure 2.

The PROMETHEE was used to:

1. **Rank methods**—for each of the subsets of questions F_0_, F_1_, F_2_, F_3_, F_4_ and F_5_ we rank the methods by their performances on that subset;
2. **Ranked subsets of questions**—for each of the models (machine-learning and statistical methods) we rank the subsets of questions by the performance of that model on them.

To run the experiments, the dataset was split into five splits and 5-fold cross-validation was used to test the performances of the models. We trained on four splits and tested on the fifth one. We repeated the same for all combinations of splits. The same splits were used for all models and all subsets for a fair comparison.

## 3. Results

### 3.1. Dimensionality Reduction

The Pearson correlation coefficients were calculated for all features. The more correlated the two features are, the higher the coefficient. Based on the calculated correlation coefficients, we chose thresholds 0.40, 0.30, 0.25, 0.20 and 0.10, and from those we got subsets of features F_1_, F_2_, F_3_, F_4_ and F_5_ respectively. As the ESFFFQ was already designed to have a minimal number of questions included, the low correlations between the questions were expected. Therefore, the thresholds were quite low. The subsets of features (number of questions and list of questions) are listed in Table 3. The full questionnaire, which we mark as set F_0_, contains 27 questions. The full questionnaire is listed in Table A1.

### 3.2. Evaluation—PROMETHEE

The results are presented in the following subsections. For each of the targets (fat intake, sugar intake, fiber intake, protein intake and salt intake) a table representing ranked methods and a table representing ranked subsets are included. In each table, the best performing method or subset is marked in bold. In tables ranking methods the results are compared column-wise and the subsets are compared row-wise.

For each subset of features the performances of the classification models were measured with precision, recall and F1-score and the regression models with mean average error (MAE), mean squared error (MSE), root mean squared error (RMSE) and coefficient of determination (R2-score). Precision, recall, F-score and R2-score are maximizing metrics (higher is better), while MAE, MSE and RMSE are minimizing metrics (lower is better). The results, ranked methods across all subsets of questions and subsets across all methods, are presented in the tables. To compare the performances of methods, elements are compared column-wise and the subsets row-wise. For better representation the best rank is marked in bold.

#### 3.2.1. Fat Intake

In Table 4 the rankings of the methods on all subsets of features for target fat intake are presented. It is obvious that the best methods to get the scores (classification) and amounts (regression) on the full subset F_0_ of questions were the statistical methods, as in this case we were not working with any missing data and the calculations were actually the ground truth. This was same for all other targets as well (sugar intake, fiber intake, protein intake and salt intake).

For the classification problem, F_0_ and F_1_ were the only subsets where statistical methods performed better than machine learning; and for the regression problem, machine learning performed better on all subsets. The overall best methods on all subsets were SVM for the classification problem and voting classifier for the regression problem.

When ranking the subsets (see Table 5), it is again obvious that the best performing subset for statistical methods “zero imputation” and “multiple imputation” was the full subset of questions F_0_. This again stands for all the targets and will not be repeated in the following subsections. For target fat intake, machine learning also worked the best on the full set of questions. For the regression problem, the same is true, except that the SVM worked the best on the subset F_1_. Additionally, it is possible to see that the performances of the kNN on F_0_ and F_1_ are very similar. However, the best subset for the regression and classification problems is the full subset of questions. Moreover, for most methods the loss of information between subsets F_0_ and F_1_ is lower than the loss of information between F_1_ and F_2_.

#### 3.2.2. Sugar Intake

As seen in Table 6, for sugar intake statistical methods worked a bit better than machine-learning algorithms, even on some of the smaller subsets, not just on F_0_. However, as the subsets got equal to or smaller than subset F_3_, statistical methods started to perform far worse and machine-learning algorithms worked better. The overall best algorithm was the gradient boosting classifier for both the classification and the regression problem.

Subset ranking (Table 7) for sugar intake shows that, contrary to fat intake, the full set of questions works the best only for statistical methods. For almost all classification and regression machine-learning algorithms, smaller subsets perform better. The overall best subset of questions for both regression and classification problems was F_2_, which included 16 questions (for the specific list of questions please refer to Table 3 and Table A1). Half of the classification algorithms and most of the regression problems performed the best on the subset F_2_.

#### 3.2.3. Fiber Intake

For fiber intake statistical methods again worked the best from all the methods on the full subset of questions (Table 8). Like for sugar intake, in this experiment the multiple imputation works best out of all methods on subsets F_1_ and F_2_ as well, both for the classification and the regression problem. For smaller subsets, the machine-learning approach takes over. The overall best methods for classification and regression problems are the gradient boosting classifier and regressor.

Rankings of subsets in Table 9 show that smaller subsets generally still give better results for the majority of the models. For most of the machine-learning algorithms for both classification and regression problems, the subsets of questions smaller or equal to F_2_ give better results.

#### 3.2.4. Protein Intake

For protein intake, statistical methods perform edthe best on the full set of questions F_0_, and for the regression problem multiple imputation performed the best on subset F_1_ as well (see Table 10). In all other cases, machine learning worked better. It is interesting that with previous targets, more complex machine learning models performed the best, while for protein intake, linear models (logistic and linear regression) performed better. For the classification problem logistic regression performed the best on three subsets out of six and also had the best overall performance. For the regression problem the best overall algorithm was again the gradient boosting regressor; however, linear regression had the second-best performance (see Table 10).

As is visible in Table 11, the overall best subset for the classification problem was F_2_. For the regression problem, the best performance was achieved on the full set of questions—except when using SVM.

#### 3.2.5. Salt Intake

For salt intake, machine-learning algorithms performed better than statistical methods. The results in Table 12 show that for the classification problem statistical methods are almost always ranked in the last two positions, except of course, for the full set of questions.

The results in Table 13 show that the overall best subset for classification and regression problems for salt intake was the full set of questions F_0_. Generally, performance got worse when removing questions. However, there were some cases wherein a method performed better on a smaller subset. For instance, the decision tree regressor performed better on F_2_ than it did on F_1_.

## 4. Discussion

From the results for each target separately, the overall best method and subset were also deduced. Note that this should not be taken as the ultimate optimal result but more as a suggestion to consider a machine-learning approach as a way to deal with missing data or a way to omit possible redundant questions.

It is expected that the statistical methods will work better than machine learning when dealing with the full questionnaire (set F_0_), as this is actually the ground truth. Predictive models based on ML do not work so well because of overfitting, which happens when a model learns the detail and noise in the training data to the extent that it negatively impacts the performance of the model on new data. However, the aim of this paper is to show that when only answers are available, the precision of the scores could be improved by using machine learning. When dealing with full questionnaires, it is obvious that the conventional calculation of amounts and scores is a more reasonable choice than the use of machine learning.

The fact that the best three methods are machine-learning algorithms (see Table 14) suggests that using a machine-learning approach when dealing with missing data could be of great value. Moreover, the fact that gradient boosting and voting are among the best three algorithms for the classification and regression problems could further indicate that using these algorithms could have the greatest benefit when only a smaller subset of answers to the questions is available. Gradient boosting is an extremely popular machine-learning algorithm that has been proven successful across many domains and is one of the leading methods for winning Kaggle competitions [18].

Table 15 shows that the best subset of questions for classification is subset F_1_. Again, the reader should note that the conclusion of this should not be that subset F_1_ could replace the original questionnaire; instead, it indicates that some of the questions are redundant and could be omitted without losing much information. To explain—by making sure that the user answers at least the questions in this subset, we could get enough information to estimate the quality of the user’s nutrition habits quite well even if he/she does not answer the remaining questions. An additional conclusion derived from the results would be that the machine-learning approach has proven itself a very useful approach and its applications could be explored further, even when working with missing data in FFQs. It outperformed the baseline approach, multiple imputation [19], when dealing with subsets of questions.

The paper shows that for the classification problem, some targets of the questions could be redundant. The best results for the classification problem for targets sugar intake, fiber intake and protein intake were achieved on smaller subsets F_1_ and F_2_, which could indicate that some of the questions are redundant for these targets.

Dimensionality reduction, as proposed in the paper, could work even better for more extensive questionnaires—it is important to point out that the full ESFFFQ was already carefully designed to cover all the goals and be short at the same time. Therefore, the correlation coefficients between questions were quite low to begin with. Additionally, when removing the questions, one of the pairs was removed by no particular criteria. This should generally should not be a problem, but as correlations were not really high in the first place, we might achieve better results by removing the other of the two correlated questions. A possible improvement would be to choose additional criteria for removing one of the two correlated questions or to choose another feature selection method, for instance, information gain or something similar.

Nevertheless, the proposed dimensionality reduction approach should be considered another contribution of this paper. Very extensive questionnaires could lead towards the overestimating of nutrient values, as shown in [3], and by reducing them through omitting redundant questions, the results could come closer to the true values. This could be validated using other nutrition monitoring approaches, for instance, 24 h recalls or laboratory tests.

Choosing the PROMETHEE for evaluation makes the whole comparison more robust—for different situations, different measures are important, and by considering more measures the results are more comprehensive.

The findings of the study could be integrated into the WellCo project in the following way. For instance, if the user does not feel like answering the whole questionnaire every 2 weeks, but would still like to get some feedback on his nutrition, the system should make sure to ask the questions starting with those included in subset F_5_, rather than adding the questions from F_4_ that are not included in F_5_, etc. When the maximum number of questions that the user is willing to answer is reached, one of the best-performing algorithms on this subset should be used (Table 16) to either predict diet quality scores or predict nutrient intake.

## 5. Conclusions and Future Work

This paper explored how the dimensionality reduction of an FFQ affects the predictions of the nutrient value amounts and diet quality scores. It compared selected machine-learning algorithms with established statistical methods, zero imputation and multiple imputation. The starting hypothesis—that machine learning will perform better than statistical methods on smaller subsets of features—was confirmed. The proposed method for dimensionality reduction provided feature sets, and the PROMETHEE method was used to rank them by performance. Although this has been done on a very specific questionnaire, the proposed approach could be used as a method for other FFQs as well. Although machine learning has proven itself as a very useful approach for optimization of FFQs, it is also important to choose the methods cautiously by using robust evaluation methods. Therefore, an additional contribution of this paper is that we proposed to use the PROMETHEE as the evaluation method for comparing methods and their performances for the optimization of FFQs.

In future work, the usage of machine learning for FFQs will be explored further. By ranking the questions by importance for each of the targets, we could easily build a smaller questionnaire specific for a chosen target. The proposed approach could be used on a more extensive questionnaire, for instance, the FFQ used in National Health and Nutrition Examination Survey (NHANES) [20], in combination with the 24 h recalls and/or laboratory tests. Predicting diet quality scores or nutrient intake based on a subset of questions would be one option; however, by using available data from 24 h recalls and laboratory tests, one could format an FFQ for a specific target. This indicates that machine learning could be considered for selecting food items when creating new questionnaires targeting a very specific goal. Machine learning has proven to overtake other methods in many other areas, and the same could happen for this problem as well.

## Figures and Tables

**Table 1:** Amounts of vegetable intake in grams for different frequencies of consumption.

Answer_tag	Rarely or Never	Less than Once a Week	Once a Week	2–3 Times a Week	4–6 Times a Week	1–2 Times a Day	3–4 Times a Day	5+ a Day
salad	0.00	4.00	11.2	28.8	56.8	120	280	480
vegetables	0.00	4.00	11.2	28.8	56.8	120	280	480

**Table 2:** Diet quality scores from the vegetable intake.

Score	1 (Bad)	2 (Medium)	3 (Good)
Vegetables	less than 80 g	80 g to 240 g	more than 240 g

**Table 3:** List of features above different thresholds.

Threshold	# of Features	Features	Feature Set Tag
0.40	19	fruit, juice, salad, vegetables, chips, beans, fiber, wholebread, cheese, cakes, cream, grains, pizza, nuts_salt, nuts, potato, readmeat, whitemeat, fish	F_1_
0.30	16	fruit, juice, salad, vegetables, chips, beans, fiber, wholebread, cheese, cakes, grains, nuts, potato, readmeat, whitemeat, fish	F_2_
0.25	10	fruit, juice, salad, beans, fiber, wholebread, grains, nuts, readmeat, whitemeat	F_3_
0.20	6	fruit, juice, salad, fiber, wholebread, grains	F_4_
0.10	2	fruit, juice	F_5_

**Table 4:** Rankings of classification and regression models for different feature sets for fat.

	Classification							Regression						
	**F_0_**	**F_1_**	**F_2_**	**F_3_**	**F_4_**	**F_5_**	**avg.**	**F_0_**	**F_1_**	**F_2_**	**F_3_**	**F_4_**	**F_5_**	**avg.**
Logistic/Linear Regression	9.0	6.0	**1.0**	5.0	4.0	4.5	4.0	7.0	6.0	3.5	2.5	**1.0**	**1.0**	2.0
K-Nearest Neighbors	10.0	8.0	5.0	2.0	7.0	**1.0**	6.0	8.0	7.0	5.0	2.5	4.0	4.0	6.0
Decision Tree	8.0	9.0	9.0	7.0	8.0	3.0	9.0	9.0	8.0	9.0	8.0	8.0	7.5	9.0
SVM	4.0	3.0	3.0	**1.0**	6.0	2.0	**1.0**	10.0	9.0	7.0	4.0	2.0	**1.0**	6.0
Bagging Clf./Reg.	7.0	7.0	8.0	8.0	5.0	7.0	8.0	6.0	5.0	6.0	6.0	7.0	7.5	7.0
Gradient Boosting Clf./Reg.	5.0	5.0	2.0	4.0	2.5	4.5	3.0	3.0	**1.0**	3.5	7.0	5.5	5.5	3.5
Random Forest	6.0	2.0	6.0	6.0	2.5	8.0	5.0	4.0	3.5	2.0	5.0	5.5	5.5	3.5
Voting Clf./Reg.	3.0	4.0	4.0	3.0	**1.0**	6.0	2.0	5.0	3.5	**1.0**	**1.0**	3.0	**1.0**	**1.0**
Zero imputation	**1.5**	10.0	10.0	10.0	10.0	9.5	10.0	**1.5**	10.0	10.0	10.0	10.0	10.0	10.0
Multiple imputation	**1.5**	**1.0**	7.0	9.0	9.0	9.5	7.0	**1.5**	2.0	8.0	9.0	9.0	9.0	8.0

**Table 5:** Rankings of feature sets for different classification and regression models for fat.

	Classification						Regression					
	**F_0_**	**F_1_**	**F_2_**	**F_3_**	**F_4_**	**F_5_**	**F_0_**	**F_1_**	**F_2_**	**F_3_**	**F_4_**	**F_5_**
Logistic/Linear Regression	**1.0**	2.0	3.0	4.0	5.0	6.0	**1.0**	2.0	3.0	5.0	4.0	6.0
K-Nearest Neighbors	**1.0**	2.0	3.0	4.0	5.0	6.0	**1.0**	2.0	3.0	4.0	5.0	6.0
Decision Tree	**1.0**	2.0	3.0	4.0	5.0	6.0	**1.0**	2.0	4.0	5.0	6.0	3.0
SVM	**1.0**	2.0	4.0	3.0	5.0	6.0	2.0	**1.0**	3.0	4.0	5.0	6.0
Bagging Classifier/Regressor	**1.0**	2.0	3.0	5.0	4.0	6.0	**1.0**	2.0	3.0	4.0	6.0	5.0
Gradient Boosting Classifier/Regressor	**1.0**	2.0	3.0	4.0	5.0	6.0	**1.0**	2.0	3.0	4.0	5.0	6.0
Random Forest	**1.0**	2.0	3.0	4.0	5.0	6.0	**1.0**	2.0	3.0	4.0	5.0	6.0
Voting Classifier/Regressor	**1.0**	2.0	3.0	4.0	5.0	6.0	**1.0**	2.0	3.0	4.0	5.0	6.0
Zero imputation	**1.0**	2.0	3.0	4.0	6.0	5.0	**1.0**	2.0	3.0	4.0	5.0	6.0
Multiple imputation	**1.0**	2.0	3.0	4.0	5.0	6.0	**1.0**	2.0	3.0	4.0	5.0	6.0
Average rank	**1.0**	2.0	3.0	4.0	5.0	6.0	**1.0**	2.0	3.0	4.0	5.0	6.0

**Table 6:** Rankings of classification and regression models for different feature sets for sugar.

	Classification							Regression						
	**F_0_**	**F_1_**	**F_2_**	**F_3_**	**F_4_**	**F_5_**	**avg.**	**F_0_**	**F_1_**	**F_2_**	**F_3_**	**F_4_**	**F_5_**	**avg.**
Logistic/Linear Reg.	8.0	8.0	8.0	5.0	5.0	8.5	8.0	8.0	8.0	8.0	**1.0**	**1.5**	**1.5**	4.0
K-Nearest Neighbors	9.0	9.0	10.0	7.0	9.0	6.0	9.0	9.0	9.0	9.0	4.0	3.0	5.0	8.0
Decision Tree	6.0	7.0	7.0	8.0	6.0	**1.0**	6.0	7.0	7.0	7.0	9.0	9.0	8.0	9.0
SVM	10.0	10.0	9.0	9.0	8.0	8.5	10.0	10.0	10.0	10.0	6.0	7.0	5.0	10.0
Bagging Clf./Reg.	4.0	4.5	4.0	2.5	**1.0**	2.0	2.0	5.0	5.0	4.0	7.0	6.0	5.0	6.0
Gradient Boost. Clf./Reg.	3.0	3.0	2.0	**1.0**	3.0	4.0	**1.0**	3.0	3.0	2.0	3.0	4.5	5.0	**1.0**
Random Forest	7.0	4.5	3.0	2.5	4.0	5.0	5.0	4.0	4.0	3.0	5.0	4.5	5.0	3.0
Voting Clf./Reg.	5.0	6.0	5.0	4.0	2.0	3.0	3.5	6.0	6.0	6.0	2.0	**1.5**	**1.5**	2.0
Zero imputation	**1.5**	2.0	6.0	10.0	10.0	8.5	7.0	**1.5**	2.0	5.0	10.0	10.0	10.0	7.0
Multiple imputation	**1.5**	**1.0**	**1.0**	6.0	7.0	8.5	3.5	**1.5**	**1.0**	**1.0**	8.0	8.0	9.0	5.0

**Table 7:** Rankings of feature sets for different classification and regression models for sugar.

	Classification						Regression					
	**F_0_**	**F_1_**	**F_2_**	**F_3_**	**F_4_**	**F_5_**	**F_0_**	**F_1_**	**F_2_**	**F_3_**	**F_4_**	**F_5_**
Logistic/Linear Regression	3.0	**1.0**	2.0	5.0	4.0	6.0	3.0	2.0	**1.0**	5.0	4.0	6.0
K-Nearest Neighbors	5.0	4.0	**1.0**	2.0	3.0	6.0	3.0	2.0	**1.0**	5.0	4.0	6.0
Decision Tree	**1.0**	2.0	3.0	5.0	4.0	6.0	3.0	**1.0**	2.0	6.0	5.0	4.0
SVM	6.0	4.0	**1.0**	3.0	2.0	5.0	3.0	2.0	**1.0**	5.0	4.0	6.0
Bagging Classifier/Regressor	**1.0**	2.0	3.0	5.0	4.0	6.0	3.0	2.0	**1.0**	5.0	4.0	6.0
Gradient Boosting Classifier/Regressor	2.0	**1.0**	3.0	5.0	4.0	6.0	2.0	**1.0**	3.0	4.0	5.0	6.0
Random Forest	3.0	2.0	**1.0**	5.0	4.0	6.0	3.0	2.0	**1.0**	5.0	4.0	6.0
Voting Classifier/Regressor	2.0	3.0	**1.0**	5.0	4.0	6.0	3.0	2.0	**1.0**	5.0	4.0	6.0
Zero imputation	**1.0**	2.0	3.0	4.0	6.0	5.0	**1.0**	2.0	3.0	4.0	5.0	6.0
Multiple imputation	**1.0**	2.0	3.0	5.0	4.0	6.0	**1.0**	2.0	3.0	4.0	5.0	6.0
Average rank	3.0	2.0	**1.0**	5.0	4.0	6.0	3.0	2.0	**1.0**	5.0	4.0	6.0

**Table 8:** Rankings of classification and regression models for different feature sets for fiber.

	Classification							Regression						
	**F_0_**	**F_1_**	**F_2_**	**F_3_**	**F_4_**	**F_5_**	**avg.**	**F_0_**	**F_1_**	**F_2_**	**F_3_**	**F_4_**	**F_5_**	**avg.**
Logistic/Linear Regression	3.0	6.5	5.0	**1.0**	4.0	7.0	2.0	7.0	7.0	7.0	5.0	**1.5**	2.0	4.5
K-Nearest Neighbors	10.0	10.0	10.0	7.0	**1.5**	4.0	9.0	8.0	8.0	8.0	6.0	3.0	4.5	7.0
Decision Tree	6.0	9.0	9.0	9.0	8.0	3.0	10.0	10.0	10.0	10.0	9.0	8.0	8.0	10.0
SVM	9.0	8.0	7.0	2.0	1.5	10.0	8.0	9.0	9.0	9.0	7.0	4.5	**1.0**	8.5
BaggingClf./Reg.	7.0	6.5	4.0	6.0	7.0	**1.0**	6.0	5.0	6.0	5.0	4.0	7.0	6.5	6.0
Gradient Boosting Clf./Reg.	4.0	3.0	3.0	5.0	6.0	2.0	**1.0**	3.0	3.0	2.0	**1.0**	4.5	4.5	**1.0**
Random Forest	5.0	5.0	8.0	3.0	5.0	5.0	5.0	5.0	4.0	4.0	2.0	6.0	6.5	3.0
Voting Clf./Reg.	8.0	4.0	2.0	4.0	3.0	9.0	4.0	5.0	5.0	3.0	3.0	**1.5**	3.0	2.0
Zero imputation	**1.5**	2.0	6.0	10.0	10.0	7.0	7.0	**1.5**	2.0	6.0	10.0	10.0	10.0	8.5
Multiple imputation	**1.5**	**1.0**	**1.0**	8.0	9.0	7.0	3.0	**1.5**	**1.0**	**1.0**	8.0	9.0	9.0	4.5

**Table 9:** Rankings of feature sets for different classification and regression models for fiber.

	Classification						Regression					
	**F_0_**	**F_1_**	**F_2_**	**F_3_**	**F_4_**	**F_5_**	**F_0_**	**F_1_**	**F_2_**	**F_3_**	**F_4_**	**F_5_**
Logistic/Linear Regression	2.0	3.0	4.0	**1.0**	5.0	6.0	**1.0**	2.0	3.0	4.0	5.0	6.0
K-Nearest Neighbors	3.0	5.0	4.0	2.0	**1.0**	6.0	2.0	**1.0**	3.0	4.0	5.0	6.0
Decision Tree	2.0	**1.0**	3.0	4.0	5.0	6.0	2.0	3.0	**1.0**	4.0	6.0	5.0
SVM	4.0	2.0	5.0	**1.0**	3.0	6.0	3.0	**1.0**	2.0	4.0	5.0	6.0
Bagging Classifier/Regressor	3.0	**1.0**	2.0	4.0	5.0	6.0	**1.0**	2.0	3.0	4.0	5.0	6.0
Gradient Boosting Classifier/Regressor	2.0	**1.0**	3.0	4.0	5.0	6.0	**1.0**	2.0	3.0	4.0	5.0	6.0
Random Forest	3.0	**1.0**	5.0	2.0	4.0	6.0	2.0	**1.0**	3.0	4.0	5.0	6.0
Voting Classifier/Regressor	5.0	**1.0**	2.0	3.0	4.0	6.0	**1.0**	2.0	3.0	4.0	5.0	6.0
Zero imputation	**1.0**	2.0	3.0	4.0	6.0	5.0	**1.0**	2.0	3.0	4.0	5.0	6.0
Multiple imputation	**1.0**	2.0	3.0	4.0	5.0	6.0	**1.0**	2.0	3.0	4.0	5.0	6.0
Average rank	2.0	**1.0**	4.0	3.0	5.0	6.0	**1.0**	2.0	3.0	4.0	5.0	6.0

**Table 10:** Rankings of classification and regression models for different feature sets for protein.

	Classification							Regression						
	**F_0_**	**F_1_**	**F_2_**	**F_3_**	**F_4_**	**F_5_**	**avg.**	**F_0_**	**F_1_**	**F_2_**	**F_3_**	**F_4_**	**F_5_**	**avg.**
Logistic/Linear Regression	3.0	**1.0**	**1.0**	**1.0**	2.0	7.5	**1.0**	4.0	3.0	2.0	3.0	3.5	**1.0**	2.0
K-Nearest Neighbors	5.0	4.0	3.0	2.0	**1.0**	4.0	2.0	8.0	7.0	7.0	6.0	3.5	7.0	7.0
Decision Tree	10.0	10.0	9.0	9.0	5.0	5.5	10.0	9.0	10.0	9.0	8.0	8.0	7.0	10.0
SVM	7.5	3.0	7.0	5.0	8.0	7.5	7.0	10.0	9.0	8.0	7.0	6.5	**1.0**	8.0
Bagging Clf./Reg.	9.0	6.0	5.5	7.0	6.0	5.5	8.0	7.0	6.0	6.0	5.0	6.5	4.5	6.0
Gradient Boosting Clf./Reg.	4.0	9.0	8.0	4.0	3.0	2.0	5.0	3.0	2.0	**1.0**	**1.0**	3.5	4.5	**1.0**
Random Forest	6.0	8.0	4.0	8.0	7.0	3.0	6.0	6.0	5.0	4.0	3.0	3.5	7.0	4.0
Voting Clf./Reg.	7.5	5.0	5.5	6.0	4.0	**1.0**	4.0	5.0	4.0	3.0	3.0	**1.0**	**1.0**	3.0
Zero imputation	**1.5**	7.0	10.0	10.0	10.0	9.0	9.0	**1.5**	8.0	10.0	10.0	10.0	10.0	9.0
Multiple imputation	**1.5**	2.0	2.0	3.0	9.0	10.0	3.0	**1.5**	**1.0**	5.0	9.0	9.0	9.0	5.0

**Table 11:** Ranking of feature sets for different classification and regression models for target protein.

	Classification						Regression					
	**F_0_**	**F_1_**	**F_2_**	**F_3_**	**F_4_**	**F_5_**	**F_0_**	**F_1_**	**F_2_**	**F_3_**	**F_4_**	**F_5_**
Logistic/Linear Regression	3.0	**1.0**	2.0	4.0	5.0	6.0	**1.0**	2.0	3.0	4.0	5.0	6.0
K-Nearest Neighbors	3.0	**1.0**	2.0	4.0	5.0	6.0	**1.0**	2.0	3.0	4.0	5.0	6.0
Decision Tree	**1.0**	3.0	2.0	6.0	5.0	4.0	**1.0**	2.0	3.0	4.0	6.0	5.0
SVM	3.0	**1.0**	2.0	4.0	6.0	5.0	3.0	2.0	**1.0**	4.0	5.0	6.0
Bagging Classifier/Regressor	2.0	3.0	**1.0**	4.0	6.0	5.0	**1.0**	2.0	3.0	4.0	5.0	6.0
Gradient Boosting Classifier/Regressor	**1.0**	4.0	2.0	3.0	6.0	5.0	**1.0**	2.0	3.0	4.0	5.0	6.0
Random Forest	2.0	3.0	**1.0**	5.0	6.0	4.0	**1.0**	2.0	3.0	4.0	5.0	6.0
Voting Classifier/Regressor	2.0	3.0	**1.0**	4.0	6.0	5.0	**1.0**	2.0	3.0	4.0	5.0	6.0
Zero imputation	**1.0**	2.0	3.0	4.0	6.0	5.0	**1.0**	2.0	3.0	4.0	5.0	6.0
Multiple imputation	**1.0**	2.0	3.0	4.0	5.0	6.0	**1.0**	2.0	3.0	4.0	5.0	6.0
Average rank	2.0	3.0	**1.0**	4.0	6.0	5.0	**1.0**	2.0	3.0	4.0	5.0	6.0

**Table 12:** Rankings of classification and regression models for different feature sets for the target salt.

	Classification							Regression						
	**F_0_**	**F_1_**	**F_2_**	**F_3_**	**F_4_**	**F_5_**	**avg.**	**F_0_**	**F_1_**	**F_2_**	**F_3_**	**F_4_**	**F_5_**	**avg.**
Logistic/Linear Regression	5.0	3.0	**1.0**	**1.0**	3.0	7.0	**1.0**	5.0	**1.0**	**1.0**	2.5	2.0	**1.5**	**1.0**
K-Nearest Neighbors	8.0	8.0	8.0	7.0	6.0	**1.0**	7.0	9.0	8.0	7.0	6.0	4.5	6.5	7.0
Decision Tree	10.0	9.0	9.0	5.5	7.0	3.0	9.0	10.0	9.0	9.0	8.0	8.0	6.5	9.0
SVM	3.0	**1.0**	2.0	3.0	8.0	8.0	3.0	6.5	6.0	6.0	6.0	6.5	**1.5**	5.0
Bagging Clf./Reg.	9.0	5.5	5.0	2.0	2.0	5.0	4.0	8.0	5.0	5.0	6.0	6.5	6.5	6.0
Gradient Boosting Clf./Reg.	6.0	3.0	6.0	5.5	5.0	4.0	5.0	3.0	2.5	3.0	2.5	2.0	4.0	3.0
Random Forest	7.0	5.5	4.0	4.0	4.0	6.0	6.0	6.5	4.0	4.0	2.5	4.5	6.5	4.0
Voting Clf./Reg.	4.0	3.0	3.0	8.0	**1.0**	2.0	2.0	4.0	2.5	2.0	2.5	2.0	3.0	2.0
Zero imputation	**1.5**	10.0	10.0	10.0	10.0	9.5	10.0	**1.5**	10.0	10.0	10.0	10.0	10.0	10.0
Multiple imputation	**1.5**	7.0	7.0	9.0	9.0	9.5	8.0	**1.5**	7.0	8.0	9.0	9.0	9.0	8.0

**Table 13:** Rankings of feature sets for different classification and regression models for salt.

	Classification						Regression					
	**F_0_**	**F_1_**	**F_2_**	**F_3_**	**F_4_**	**F_5_**	**F_0_**	**F_1_**	**F_2_**	**F_3_**	**F_4_**	**F_5_**
Logistic/Linear Regression	**1.0**	3.0	2.0	4.0	5.0	6.0	**1.0**	2.0	3.0	4.0	5.0	6.0
K-Nearest Neighbors	**1.0**	2.0	3.0	4.0	5.0	6.0	**1.0**	2.0	3.0	4.0	5.0	6.0
Decision Tree	**1.0**	2.0	3.0	4.0	5.0	6.0	**1.0**	3.0	2.0	5.0	6.0	4.0
SVM	**1.0**	2.0	3.0	4.0	5.0	6.0	**1.0**	2.0	3.0	4.0	5.0	6.0
Bagging Classifier/Regressor	**1.0**	2.0	3.0	4.0	5.0	6.0	**1.0**	2.0	3.0	4.0	5.0	6.0
Gradient Boosting Classifier/Regressor	**1.0**	2.0	3.0	4.0	5.0	6.0	**1.0**	2.0	3.0	4.0	5.0	6.0
Random Forest	**1.0**	2.0	3.0	4.0	5.0	6.0	**1.0**	2.0	3.0	4.0	5.0	6.0
Voting Classifier/Regressor	**1.0**	2.0	3.0	5.0	4.0	6.0	**1.0**	2.0	3.0	4.0	5.0	6.0
Zero imputation	**1.0**	2.0	3.0	6.0	5.0	4.0	**1.0**	2.0	3.0	4.0	5.0	6.0
Multiple imputation	**1.0**	2.0	3.0	4.0	5.0	6.0	**1.0**	2.0	3.0	4.0	5.0	6.0
Average rank	**1.0**	2.0	3.0	4.0	5.0	6.0	**1.0**	2.0	3.0	4.0	5.0	6.0

**Table 14:** Overall ranking of classification and regression models for different feature sets.

	Classification	Regression
	**Average Rank**	**Average Rank**
Logistic/Linear Regression	5.0	6.0
K-Nearest Neighbors	9.0	7.0
Decision Tree	7.0	9.0
SVM	8.0	8.0
Bagging Classifier/Regressor	**1.0**	4.0
Gradient Boosting Classifier/Regressor	**3.0**	**1.0**
Random Forest	6.0	**3.0**
Voting Classifier/Regressor	**2.0**	**2.0**
Zero imputation	10.0	10.0
Multiple imputation	4.0	6.0

**Table 15:** Overall rankings of feature sets for different classification and regression models.

	Classification						Regression					
	**F_0_**	**F_1_**	**F_2_**	**F_3_**	**F_4_**	**F_5_**	**F_0_**	**F_1_**	**F_2_**	**F_3_**	**F_4_**	**F_5_**
Average rank	2.0	**1.0**	3.0	4.0	5.0	6.0	**1.0**	2.0	3.0	4.0	5.0	6.0

**Table 16:** Rankings of different classification and regression models for each subset for all targets.

	Classification						Regression					
Logistic/Linear Regression	7.0	**2.0**	3.0	**1.0**	5.0	7.0	7.0	6.0	6.0	3.0	2.0	**1.0**
K-Nearest Neighbors	10.0	10.0	10.0	6.0	6.0	4.0	9.0	8.0	8.0	5.0	3.0	5.5
cDecision Tree	8.0	8.0	7.0	9.0	7.0	**1.0**	8.0	9.0	7.0	9.0	9.0	8.0
SVM	9.0	9.0	9.0	7.0	8.0	8.0	10.0	10.0	10.0	7.0	6.5	3.0
Bagging Classifier/Regressor	6.0	5.0	4.0	3.0	2.0	2.0	6.0	5.0	5.0	6.0	6.5	5.5
Gradient Boosting Classifier/Regressor	3.0	4.0	5.0	2.0	3.0	3.0	3.0	2.0	**1.0**	**1.5**	4.0	4.0
Random Forest	5.0	6.0	6.0	5.0	4.0	6.0	4.0	3.0	3.0	4.0	5.0	7.0
Voting Classifier/Regressor	4.0	3.0	2.0	4.0	**1.0**	5.0	5.0	4.0	4.0	**1.5**	**1.0**	2.0
Zero imputation	**1.5**	7.0	8.0	10.0	10.0	9.0	1.5	7.0	9.0	10.0	10.0	10.0
Multiple imputation	**1.5**	**1.0**	**1.0**	8.0	9.0	10.0	**1.5**	**1.0**	2.0	8.0	8.0	9.0

## Appendix A

**Table A1:** List of questions and features in the ESFFFQ.

Question	Feature
How often do you eat fruit (tinned/fresh)?	fruit
How often do you drink juice (not cordial or squash)?	juice
How often do you eat salad (not garnish or added to sandwiches)?	salad
How often do you eat vegetables (tinned/frozen/fresh, but not potatoes)?	veg
How often do you eat chips/fried potatoes?	chips
How often do you eat beans or pulses (baked beans, chick peas, dahl…)?	beans
How often do you eat fiber-rich breakfast cereal (porridge, muesli…)?	fiber
How often do you eat wholemeal bread or chapattis?	wholebread
How often do you eat cheese/yogurt?	cheese
How often do you eat crisps/savoury snacks?	crisps
How often do you eat sweet biscuits, cakes, chocolate, sweets?	cakes
How often do you eat ice cream/Cream?	cream
How often do you drink non-alcoholic fizzy drinks/pop (not sugar free or diet?	pop
How often do you eat **SALTED** nuts, peanuts or seeds?	nuts_salted
How often do you eat **UNSALTED** nuts, peanuts or seeds?	nuts
How often do you eat grains (pasta, rice, couscous, bulgur…)?	grains
How often do you eat pre-prepared sauces, gravies, dry soup mixes?	pre-prepared
How often do you eat pizza, pasta/noodle dishes with cheese sauce?	pizza
How often do you eat bread, buns and other bread pastries (non-sweet)?	bread
How often do you eat potato (mashed, baked, cooked; not fried)?	potato
How often do you eat eggs (boiled, fried, scrambled,…)?	eggs
How often do you eat beef, lamb, pork, ham (steaks, roasts, joints, chops…)?	redmeat
How often do you eat chicken, turkey (steaks, roasts…- not in batter/breadcrumbs)?	whitemeat
How often do you eat sausages, bacon, corned beef, meat pies/pasties…?	sausage
How often do you eat chicken, turkey (nuggets/twizzlers, pies or in batter/breadcrumbs)?	nuggets
How often do you eat white fish in batter or breadcrumbs?	batterfish
How often do you eat white fish **NOT** in batter or breadcrumbs or oily fish (salmon, tuna)?	fish

## References

[1] Reščič N., Valenčič E., Mlinarič E., Seljak B.K., Luštrek M. (2019). Mobile Nutrition Monitoring for Well-Being. UbiComp/ISWC ’19 Adjunct, Proceedings of the 2019 ACM International Joint Conference on Pervasive and Ubiquitous Computing and Proceedings of the 2019 ACM International Symposium on Wearable Computers.

[2] Cleghorn C.L., Harrison R.A., Ransley J.K., Wilkinson S., Thomas J., Cade J.E. (2016). Can a dietary quality score derived from a short-form FFQ assess dietary quality in UK adult population surveys?. Public Health Nutr..

[3] Thompson F., Byers T. (1994). Dietary Assessment Resource Manual. J. Nutr..

[4] Shim J., Oh K., Kim H. (2014). Dietary assessment methods in epidemiologic studies. Epidemiol. Health.

[5] Gerdessen J.C., Souverein O.W., van‘t Veer P., de Vries J.H. (2015). Optimising the selection of food items for FFQs using Mixed Integer Linear Programming. Public Health Nutr..

[6] Panaretos D., Koloverou E., Dimopoulos A.C., Kouli G.M., Vamvakari M., Tzavelas G., Pitsavos C., Panagiotakos D.B. (2018). A comparison of statistical and machine-learning techniques in evaluating the association between dietary patterns and 10-year cardiometabolic risk (2002–2012): The ATTICA study. Br. J. Nutr..

[7] Uemura H., Ghaibeh A., Katsuura-Kamano S., Yamaguchi M., Bahari T., Ishizu M., Moriguchi H., Arisawa K. (2017). Systemic inflammation and family history in relation to the prevalence of type 2 diabetes based on an alternating decision tree. Sci. Rep..

[8] Gjoreski M., Kochev S., Reščič N., Gregorič M., Eftimov T., Seljak B.K. Exploring Dietary Intake Data collected by FPQ using Unsupervised Learning. In Proceedings of the 2019 IEEE International Conference on Big Data.

[9] Chin E.L., Simmons G., Bouzid Y.Y., Kan A., Burnett D.J., Tagkopoulos I., Lemay D.G. (2019). Nutrient Estimation from 24-Hour Food Recalls Using Machine Learning and Database Mapping: A Case Study with Lactose. Nutrients.

[10] Gregorič M., Blaznik U., Delfar N., Zaletel M., Lavtar D., Koroušić-Seljak B., Golja P., Zdešar Kotnik K., Pravst I., Fidler Mis N. (2019). Slovenian national food consumption survey in adolescents, adults and elderly: External scientific report. EFSA Support. Publ..

[11] Zupanič N., Hristov H., Gregorič M., Blaznik U., Delfar N., Seljak B., Ding E., Fidler Mis N., Pravst I. (2020). Total and Free Sugars Consumption in a Slovenian Population Representative Sample. Nutrients.

[12] Sim J., Lee J., Kwon O. (2015). Missing Values and Optimal Selection of an Imputation Method and Classification Algorithm to Improve the Accuracy of Ubiquitous Computing Applications. Math. Probl. Eng..

[13] Azur M., Stuart E., Frangakis C., Leaf P. (2011). Multiple Imputation by Chained Equations: What is it and how does it work?. Int. J. Methods Psychiatr. Res..

[14] van Buuren S. (2007). Multiple imputation of discrete and continuous data by fully conditional specification. Stat. Methods Med Res..

[15] Muller A., Mufcller A., Guido S. (2018). Introduction to Machine Learning with Python: A Guide for Data Scientists.

[16] Eftimov T., Kocev D. Performance Measures Fusion for Experimental Comparison of Methods for Multi-label Classification. Proceedings of the AAAI Spring Symposium: Combining Machine Learning with Knowledge Engineering.

[17] Brans J.P., Vincke P. (1985). A Preference Ranking Organisation Method: (The PROMETHEE Method for Multiple Criteria Decision-Making). Manag. Sci..

[18] KAGGLE. https://github.com/dmlc/xgboost/blob/master/demo/README.md#usecases.

[19] Ichikawa M., Hosono A., Tamai Y., Watanabe M., Shibata K., Tsujimura S., Oka K., Fujita H., Okamoto N., Kamiya M. (2019). Handling missing data in an FFQ: Multiple imputation and nutrient intake estimates. Public Health Nutr..

[20] NHANES. http://www.cdc.gov/nchs/nhanes/index.htm.

